# Stakeholders' perspectives on green building rating: A case study in Indonesia

**DOI:** 10.1016/j.heliyon.2019.e01328

**Published:** 2019-03-20

**Authors:** Mohammed Ali Berawi, Perdana Miraj, Retno Windrayani, Abdur Rohim Boy Berawi

**Affiliations:** a Department of Civil Engineering, Faculty of Engineering, Universitas Indonesia, Depok, 16424 Indonesia; b Department of Civil Engineering, Faculty of Engineering, Universitas Pancasila, DKI Jakarta, 12640 Indonesia; c Center for Sustainable Infrastructure Development, Universitas Indonesia, Depok, 16424 Indonesia

**Keywords:** Civil engineering, Environmental science

## Abstract

Green building rating system has been used by many countries around the globe to certify the green buildings. However, little evidence has confirmed that stakeholders understand the theory and practical implementation of green building rating system, particularly in Indonesia. Thus, the paper aims to evaluate stakeholders knowledge of green buildings, and to propose recommendation for green building direction for the government. The result shows most of the professionals in this sector prefer considering environmental issues as the primary concern in developing the new building over social and economic aspects of sustainability. Despite their awareness of this concept, limited experience from building owner and rewards are the two main barriers to the steady progress of professionals taking certification of green building. This study also shows that the government and green building council of the country needs to evaluate the method to promote the use of green building concept and to encourage professionals taking the certification.

## 1 Introduction

The building sector has progressively become the most significant contributor to carbon emission. In the United States, buildings contribute for about 40% of national greenhouse gas emission and surpass both industrial and transportation sectors (USGBC, 2016). The US. Energy Information Administration (2012), argue that energy sources used in commercial buildings are consist of electricity (61%), natural gas (32%), district heat (5%) and fuel oil (2%). Highest emission mostly from the OECD members, but the growth is limited. On the other hand, developing countries mainly located in Asia experience significant growth and reduce the gap to OECD countries (Lucon et al., 2014).

Policies and regulations have been formulated in the past three decades through an environmentally-friendly concept in the building to combat the increasing amount of carbon emission (Trencher et al., 2016). Primarily, the concept is drawn from intensive discussion and debates on minimizing the adverse effect of buildings on the environment by improving efficiency and moderating the use of materials, energy, and spatial development.

Green building is one of the concepts to encourage the use of environmental friendly approach in the construction sector. It can be measured using specific assessments such as ecological, social, and economic. Kibert (2016), generates criteria of commercial quality from reduction of life cycle cost to the preservation of economic value. The commercial quality of buildings becomes critical as this industry involves enormous investment and labor. Despite its advantages, stakeholders such as owners and developers in Indonesia hesitate to implement the concept into their project development due to higher cost and limited knowledge of the sustainable practice. The cost of green building in the country range from 10%-15% higher than conventional building (Caesario, 2016). The additional cost is from a sophisticated design, green materials, and more advanced technologies (Zhang et al., 2011; Hwang and Tan, 2012). Sertifikasi Bangunan Hijau (2017) published buildings with a green certificate (silver, gold, and platinum) in Indonesia, and the result shows only nineteen new buildings and ten existing buildings are certified green. Furthermore, only 31 persons of the country that hold GREENSHIP professionals (GBCI, 2017).

Researcher and academics have been discussed green building from technical, financial, environmental to social perspectives (Hill and Bowen, 1997; Medineckiene et al., 2015; Shad et al., 2017). However, little evidence has confirmed that stakeholders understand the theory and practical implementation of sustainability and green building rating, particularly in Indonesia. Thus, the paper aims to evaluate stakeholders knowledge of green buildings, and to propose recommendation for green building direction for the government.

## 2 Theory

### 2.1 Sustainability in construction

Sustainability has been interpreted as an interaction, collaboration, and integration of present action to preserve natural resources for future direction and next-generation needs. The search for a sustainable way to mitigate ecological degradation and energy saving was first raised in the Brundtland Report by the United Nations World Commission on Environment and Development (WCED) in 1987, and conference of parties (COP) started in 1995 which lasts to date. The annual meetings have produced significant policies to combat environmental problems from Kyoto Protocol in 1997 to Paris Agreement in 2015 (Oberthür and Ott, 1999; Rogelj et al., 2016).

**Fig. 1:**
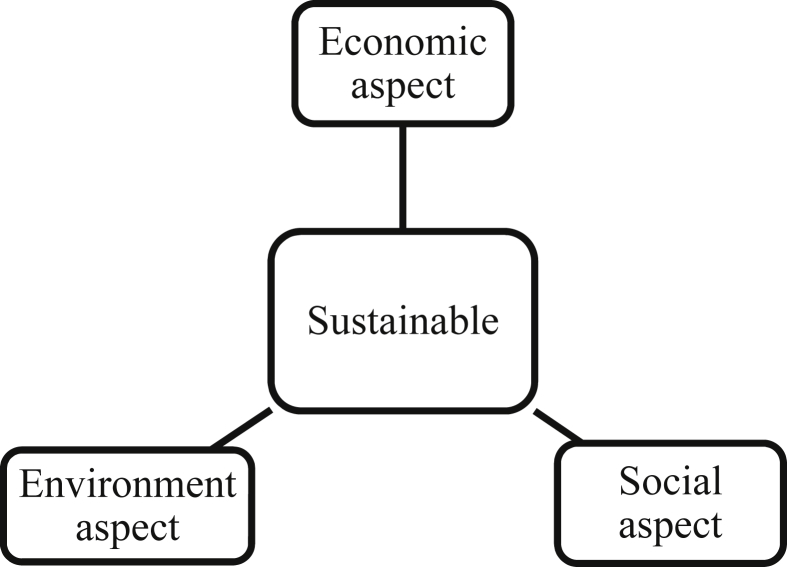
Sustainability concept.

Construction practices comprehend sustainability as a way for construction sector contributing to environmental protection. Although the term of sustainability keeps evolving through time, the basic concept lies in three main pillars; environmental, economic and social aspects, as shown in Fig. 1 (Kibwami and Tutesigensi, 2016). The environment in sustainable construction relates to the management of waste, emissions, use of renewable energy, toxic – free materials, and many others (Shi et al., 2013). Those are used to reduce the adverse effects of human activity and to expand the quality of life of human being.

Environmental concern also sees as a designer way to create buildings with eco-labeling performance. A building owner may wish to generate significant revenue from the building in particular period, while the users expect a comfort, healthy, and safety building (Ding, 2008). Minimizing the gap between the two parties expectation may generate through a sustainability concept. Sustainable construction not only offer a low operational cost for the building owner but the concept also propose a high quality of indoor health, air quality, visual and security (Shi et al., 2013; Trencher et al., 2016).

The social aspect is subjected to the concept of the human itself, the influence of civilization (Edum-Fotwe and Price, 2009), and the quality and comfort of livelihood (Dempsey et al., 2011). For instance, environment intervention and workspace configuration potentially affect social contact. Open plan office layout may be selected to provide better communication among employee and offer greater solar penetration to the central building due to minimum partitions (Kim and De Dear, 2013). Enclosed plan for the footprint can be used when privacy is the primary concern, and energy savings may generate from passive (building envelope, glazing, doors, windows and others) or active (building automated system) approaches (Sadineni et al., 2011; Henze et al., 2008).

A combination of environmental awareness and social interaction may improve the economic aspect of society (Pugh, 2014). Tan et al. (2011) illustrated two phenomena that link environment with economic success. A traditionalist where environment sees as a burden, an additional cost and more extended payback period. On the other hand, revisionist proposes environment as a trigger for competitive advantages to companies. Innovation and new technologies may be used to gain interest and becomes the pioneer to generate new market (Porter and Van der Linde, 1995). Although this concept is still debatable among practitioners and academics, many believed that the idea of sustainability and its components is the critical success factor in improving the competitiveness level of a nation (Berawi et al., 2016; Balkyte and Tvaronavičiene, 2010).

Sustainable construction is related to a design that wisely considers its impact on the environment (Weingaertner and Moberg, 2014). It aims to minimize the negative impact by improving efficiency and applying selected material, energy and space arrangement. As the building we shape today will affect the future development, awareness of sustainability has to be fostered promptly. It requires commitment from related stakeholders in the planning phase of a building project. The use of renewable energy, modern and environmentally-friendly technology should be integrated into the practice of drafting the project.

### 2.2 Green building

Green building rose during the oil crisis in 1970 and has developed exponentially due to sustainability issues in the use of non – renewable materials, greenhouse gas emission, water scarcity not only for the urban community but also rural areas and many others (Ramírez-Villegas et al., 2016; Aktas and Ozorhon, 2015). To date, the concept has been used as necessary approach around the world to support the earth and to mitigate environmental degradation.

Literature defines green building in the construction sector in many ways. Cassidy (2003) argued that green building is an attempt to increase building efficiency by considering natural resources and at the same time, improve human settlement during the building life cycle. International Initiative for a Sustainable Built Environment stated that green building is a building that has high performance with critical parameters including low energy consumption and emissions, low ecological impacts, and achieved indoor environments. The United States Environmental Protection Agency (2016) sees that green building is a development of high-performance building design that mainly considers the energy and water usage to combat environmental degradation, climate change, and natural resource reduction. Robichaud and Anantatmula (2010) define the green building as a practice to reduce the negative impact on the earth, enhance people wellbeing, improve economic development and propose public prosperity. From that definition, the ultimate goals of the green building are both to accommodate sustainability concept by integrating environment, the social and economic value of buildings and to preserve human wellbeing for the very future.

It is true that green buildings may consume higher initial cost in the initial phase rather than conventional buildings. However, the operation and maintenance shall be lower and in the longer term will recover those preliminary costs. Green buildings may reduce the operational costs range about 8–9%, improve the value of the building for more than 7.5% and increase the occupancy rates by 3.5% (Robichaud and Anantatmula, 2010). Although the paper is not focusing on the associated cost of the project, knowing the benefit of the green building will support the argument to expand such concept.

### 2.3 Environmental assessment model in building

Assessment of a building to be categorized as the green building has been attempted by many academics and researchers in the past decades. One way is by using a multi-criterion tool to evaluate building performance (Medineckiene et al., 2015; Shad et al., 2017). Many countries have developed their assessment tools and criteria. Building Research Establishment Environmental Assessment Method (BREEAM) from the UK is argued as the first method in calculating the rating of green buildings since 1988 (Schwartz and Raslan, 2013; Mollaoglu et al., 2016). An independent third-party rating system evaluates the selection from project stakeholders from concept to completion phase. On the other hand, Leadership in Energy and Environmental Design (LEED) from US Green Building Council (USGBC) explores sustainable implementation during building life cycle before the rating (Chokor et al., 2015; Kim et al., 2017). A similar concept was also found in other countries such as Green Star rating tools in Australia, ITACA in Italy (Asdrubali et al., 2015), Comprehensive Assessment System for Building Environmental Efficiency (CASBEE) in Japan, Building Environmental Assessment Methods (BEAM) in Hong Kong, and Green Building Index in Malaysia to Green Mark in Singapore (Zuo and Zhao, 2014).

Indonesia also has a rating system called GREENSHIP from Green Building Council Indonesia (GBCI) – an independent institution to certify green building in Indonesia. Unlike in the neighboring countries such as Singapore, GBCI formulates the GREENSHIP without proper support from the government regarding regulation and policy. In Singapore, Green Mark was developed by Building Construction Authority (BCA) and fully supported by the National Environment Agency (Wu and Low, 2010). The rating development by GBCI is then supported by the World Green Building Council, based in Toronto, Canada. They also elaborate the concept with technical advisory and participants from associations, universities, contractors, developers and many others. The rating contains points from the aspect of assessment, and each item has credit points. The item of criteria includes appropriate site development, energy efficiency & conservation, water conservation, material resources & cycle, indoor air health & comfort and building environment management. In total, 77 credits and five bonus credits are available for the final assessment of the new building project. The detail can be seen in Table 1.

**Table 1:** GREENSHIP category and its weighting score.

No	Rating Criteria	Maximum Points Available	Bonus
1	Appropriate Site Development	17	
2	Energy Efficiency & Conservation	26	5
3	Water Conservation	21	
4	Material Resources & Cycle	2	
5	Indoor Air Health & Comfort	5	
6	Building Environment Management	6	
	Total	77	5

Nowadays, GREENSHIP consists of four categories of certification ranging from new building rating tool, existing building rating tool, interior space, homes, and neighborhood. Nineteen buildings are certified as New Building, ten buildings registered as green for the Existing Buildings, and two interior space are also certified (Sertifikasi Bangunan Hijau, 2017).

The number of involvement in buildings during the past seven years is deemed relatively small compared to the new construction buildings in Jakarta and with the existing building spreading throughout the city. The needs to find out about limited application of buildings should be conducted to improve the use of the green building in the nation.

## 3 Methodology

**Fig. 2:**
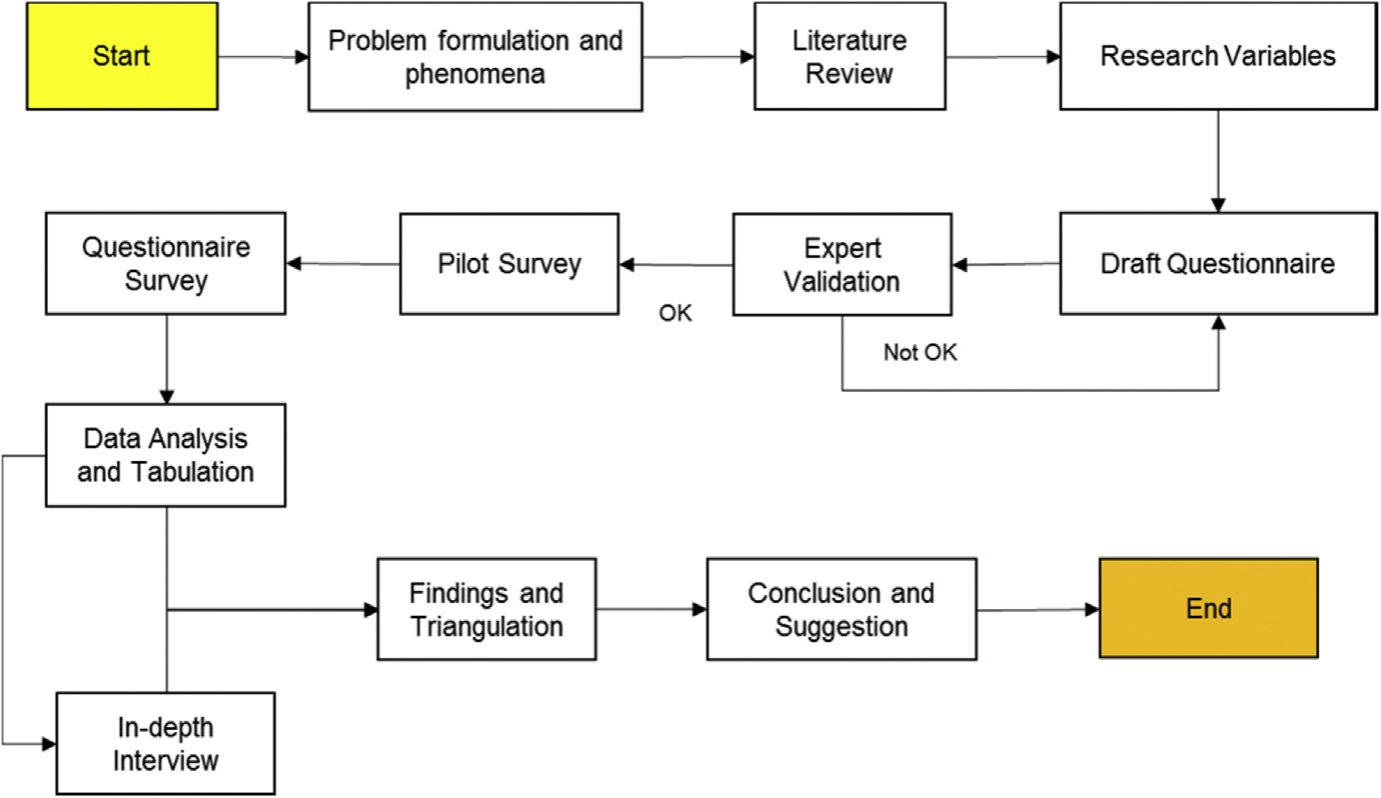
Research design.

This research combined qualitative and quantitative approach to achieve research objectives and follows research design as shown in Fig. 2. The quantitative approach uses structured instruments to generate numerical data (Berawi et al., 2017). The analysis follows statistical technique to reduce, organize, identify and determine the relationship between data (Creswell, 2013; Rahman et al., 2018). In this paper, the instruments were organized systematically to form questionnaire survey. The variables then verified and validated by five experts from academics and practitioners. They have a minimum a master degree and five-year experience in sustainable buildings. The revised version of draft questionnaire then distributed to a small scale population consists of different academics and practitioners for pilot survey. The result was used for final questionnaire instrument.

The questionnaire survey consists of four parts, namely respondents' background, awareness of sustainability, level of experience, and knowledge of green building rating.

Knowledge of the green building comprises two types of questions. First, their knowledge to determine sub-criteria in green building rating and their perspectives of cost efficiency that may generated from each criterion. Elements of cost should be selected sequentially based on their level of importance ranging from energy, operation, and renewals, water, non – fuel operational, investment and salvage value. The score of 1 means that the criteria are closely related to the element of cost and in contrast, a score of 6 means the criteria are least important. The lowest total score determines the most significant elements in green building related to cost efficiency. The example of scoring shows in Table 2.

**Table 2:** Example of scoring from the criteria to cost.

Respondent	Investment	Energy	Water	Non – Fuel Operational	Operation & Renewals	Salvage Value
R1	6	1	5	2	4	3
R2	1	6	5	4	2	3
R3	1	3	5	4	2	6
R4	6	1	2	4	6	5
R5	6	4	1	3	2	5

The questionnaire was distributed to key stakeholders using purposive sampling technique. These stakeholders should represent the population in building industry and consist of academics, consultants, contractors, developers, and officials in government institutions. All of them are located in greater Jakarta area (Jakarta, Tangerang, Bekasi, Bogor, and Depok). The respondents were selected based on their educational background, role in the project, level of experience, and type of organization they belong to. Only local respondents were selected for the questionnaire and in-depth interview to gain uniformity and to avoid cultural difference between foreigners and domestic participants (Low et al., 2015). As the questionnaire used a multiple choice approach, a descriptive analysis was selected to present the result of the returned questionnaire.

Qualitative approach examines the perspective of participants using interactive and flexible strategies. The qualitative research aims to comprehend social phenomena from the perspective of the participants (Greene and Caracelli, 1997; Abdul-Rahman and Berawi, 2002). The capability of the researchers and their interaction with respondents are critical to generating key findings related to the study (Patton, 2005). This research used an in-depth interview through a set of open-ended questions and invited three key interviewees from associations, academics, and government institutions. In-depth interview aims to validate the findings from the questionnaire survey and gain consensus from both approaches. The detail of each interviewees can be seen on Table 3.

**Table 3:** Detail of interviewees.

Interviewee	Occupancy	Level of Education	Role	Experience (Years)
A	Association	Master Degree	Manager	17
B	Practitioners	Master Degree	CEO	15
C	Lecturer	Doctoral Degree	Professor in Building Management	22

## 4 Results & discussion

### 4.1 Data collection

Out of 53 questionnaires distributed through the offline method and sent to key stakeholders in building industry, 77.36% was returned within two months of the survey. The background of respondents consists of four categories; occupation, education, role in the institution and experience. The result shows that 34.1% works as academicians, 26.8% works as private consultants while 19.5% works as contractors. With such diversity of occupation, the result expected to accommodate all related stakeholders in the building sector. Regarding education, most of them (56.1%) hold a master's and doctoral degree. Architects and project managers contribute about 46.4%, and 22% of overall respondents have more than 15 years of experience. Demographic of respondents can be seen in Table 4.

**Table 4:** Demographics of respondents.

Occupation	Frequency	Percentage (%)	Respondent Role	Frequency	Percentage (%)
Developer	2	4.90	Project Manager	4	9.80
Consultant	11	26.80	Architect	15	36.60
Contractor	8	19.50	Researcher	14	34.10
Research Institution	14	34.10	Director	4	9.80
Construction Management	2	4.90	Building evaluator	4	9.80
Government Institution	4	9.80	Total	41	100.00
Total	41	100.00			

Level of Education	Frequency	Percentage (%)	Years of Experience in the institution	Frequency	Percentage (%)
Undergraduate	18	42.00	1–8	26	63.40
Postgraduate	23	56.00	9–12	6	14.60
Total	41	100	>13	9	22.00
			Total	41	100

### 4.2 Awareness of sustainability

Awareness of sustainability consists of the purpose of sustainable design, the basic concept and the relation of project life cycle to sustainable design rating (Chokor et al., 2015; Kim et al., 2017). In this section, the respondents might select more than one answer related to the components. Regarding the purpose of sustainable design, most respondents (90.2%) select environmental protection as the main issue. Other dominant variables are natural resources protection (80.5%) as well as people's comfort and health in building (73.2%). The details are shown in Table 5.

**Table 5:** The purpose of sustainable design.

Components	Respondent Response	Percentage
Environmental protection	37	90.2%
Natural resources protection	33	80.5%
Life cycle cost reduction	26	63.4%
Preservation of economic value	17	41.5%
Comfort and health in building	30	73.2%
Preservation of social value	21	51.2%

Most of the respondents only pointed out environmental issues and overlooked other significant aspects that contribute to sustainability such as social feature and economic aspect. The result from in-depth interview shows that other aspects mostly excluded due to limited socialization from the authorities, limited knowledge of sustainability, or limited opportunities given by the project owner in developing sustainability concept to the real world.

On the other hand, there are tools to measure the environmental performance of a building using rating systems such as Leadership in Energy and Environmental Design (LEED) in the US, Green Mark in Singapore, Green Star in Australia, GREENSHIP in Indonesia and others (Chokor et al., 2015; Kim et al., 2017; Zuo and Zhao, 2014). Interviewee A argued that few of them might be suitable to be applied in specific countries yet failed to be implemented in some others due to differences in regulation and bureaucracy. Thus, it becomes crucial to evaluate rating system and put the national context in a suitable implementation.

Despite the gap of each rating system, the basic concept uses similar foundation. According to GREENSHIP rating from Green Building Council Indonesia, it consists of appropriate site development (ASD); energy efficiency and refrigerant (EER); water conservation; material resources and cycle; indoor air health and comfort; and building and environmental management. The respondents' responses are shown in Table 6.

**Table 6:** Basic concept of sustainability.

Components	Respondents' Response	Percentage
Land use	27	65.9%
Energy efficiency	37	90.2%
Water conservation	29	70.7%
Material resources and cycle	26	63.4%
Indoor air health and comfort	27	65.9%
Building and environment management	28	68.3%

The dominant factors in the basic concept of sustainability are energy efficiency (90.2%) and water conservation (70.7%). The response seemed to follow visual and quantitative measurement. Energy efficiency can be seen from the use of electricity when upper threshold exceeds then it would be declared an energy waste. Water conservation also experiences a similar situation. Other components can also be quantified although they require more complex estimation and thus they might be excluded from respondent selection due to the limited amount of time.

The reduction of energy consumption and environmental protection was also considered as the primary concern in terms of association of LCE and sustainability design. Both of the components were selected as the dominant factors with 90.2% and 73.2% respectively. More detailed results of LCE and sustainability design correlation are shown in Table 7.

**Table 7:** The correlation of LCE and sustainability design.

Components	Respondent Response	Percentage
Reduce energy consumption and material resources	37	90.2%
Maintain material cycle	24	58.5%
Environmental protection	30	73.2%
Optimum construction cost and operation	26	63.4%
Increase recycle	26	63.4%
Improve technical and functional quality	24	58.5%

According to in-depth interviews, interviewee A from the association and interviewee B from regulator argued that many people have been aware of the idea of sustainability. However, partial knowledge about the concept is the significant barrier to comprehensively implementing this. It is crucial to provide a systematic set of courses at the university with the necessary knowledge and practical guide in delivering sustainability concept. Interviewee C from academics supported the theory that architectural planning in the university tends to be applied in consideration of physical of the building, energy efficiency, and environmental issues. Social and economic aspects are not usually taken into account in the planning stage.

In terms of energy and water as the dominant factors in sustainability concept, interviewee B argued that those components consume large amounts of natural resources. Since non – renewable energy is limited and substituting it directly with renewable energy is not an option, the best strategy to reach sustainability is by minimizing the use of energy. On the other hand, all interviewees in an in-depth interview declared water as the most significant factor in the quality of life. Interviewee A claimed that Jakarta as the capital city of Indonesia consumes 150 L/person/day and should be reduced significantly to avoid land subsidence and water shortage in the future. However, water consumption figure of Jakarta is not significantly different to other neighboring countries such as Singapore, Malaysia, and Thailand. For instance, Singapore's per capita water consumption in 2015 is about 151 liters (Singapore Government, 2017), Greater Kuala Lumpur in Malaysia consume approximately 288 liters per day (Bari et al., 2015), and Bangkok is about 219.5 liters per person per day (Bangkok Post, 2015). In general, United Nations (2014), recommends a 50–100 liters per person per day to ensure basic needs and health concern for the people. The figure may vary when income level is also being considered.

In the context of Jakarta, as water management is partially operated by the government, people can freely exploit water using deep–water pump, consumption can exceed the normal volume. Water conservation is then required to maintain water availability, sustain ground level from land subsidence, and reduce floods during rainy season.

### 4.3 Level of experience

In this section, the experience of the respondents in developing a sustainable design is presented. Three main questions comprising involvement in the type of building, contribution in sustainable planning and design, and type of certificate of sustainable design were asked. The results show that 53.65% of the respondents have been involved in a sustainable design process. Most of them have experience in developing low story building (31.70%) maximum up to 3 stories. From all respondents, only 17.1% respondents have green building certificate from Green Building Council Indonesia (GBCI). The detail can be seen in Table 8.

**Table 8:** Response to the level of experience in conducting sustainable design.

Involvement in Sustainable Building	Response	Percentage (%)	Constructed building category	Response	Percentage (%)
Yes	18	43.90	Low – rise (1-3 stories)	13	31.70
No	23	56.10	Medium – rise (4–12 stories)	9	21.95
Total	41	100.00	No response	18	46.35
				41	100.00

Certificate of green building	Response	Percentage (%)
Yes	7	17.10
No	34	82.90
Total	41	100.00

According to interviewee A, the relatively low participation of national practitioners in taking green certification was caused by several factors, and one of them is the limited opportunities to elaborate green building concept into reality. The application of this rating is also a voluntary – based evaluation, thus, no obligation is required for related parties to use this concept in their building. In fact, green building expands mostly in major cities such as Jakarta, Tangerang, Solo and few others. GBCI (2017) published that out of nineteen certified new buildings and ten certified existing buildings, ten buildings and nine buildings located in Jakarta respectively.

The need to use the environmentally-friendly approach in building industry shall be supported by human resources. Professionals in Indonesia have awareness in green building concept, but only a few of them have the physical and technological experience in it. It is, therefore, become a crucial issue that must be solved by related stakeholders from the government, academics, practitioners, and owners.

From the registered project particularly in new building certification, more than 40 projects were offices, while others are hotels, apartments, library, mall, church, university, hospital and government building (GBCI, 2017). It is shown that the office building owner realizes that the use of the green building can provide significant benefits to them mainly in reducing operational and maintenance costs which in the longer term will increase their profit from the building and improve their reputation as users of green building concept.

Lastly, the government shall actively involve through strategic policy and regulation. In 2015 through Ministerial Regulation of Public Works and Housing No 02/PRT/M/2015 the government aims to generate more sustainable buildings consistently. However, interviewee C from academics claimed that the impact is insignificant. It because the implementation still limited and the number of experts in green building rises steadily. A breakthrough in decision making is required to boost the use of this concept. The incentive is one potential policy that might contribute to the implementation of green building. The concept requires higher initial cost than the conventional approach. With current practice where the lowest bidder has the opportunity to win a tender, green building application, particularly in public buildings, is far from feasible. The incentive will attract investors to deal with green building concept without compromising their overall profit.

### 4.4 Comparison of knowledge in green building components

The concept design in this section adopts six rating items from Green Building Council Indonesia (GBCI). Each item has criteria that can be quantified to evaluate the overall building performance and measure its sustainability level. The result will show the stakeholders perspective and preference in green buildings rating in Indonesia.

The respondent responses are processed through the SPSS software. It generates the number of response, percentage of response and percent of cases. There are two percentages that showed by the software. Percentage of response is response of a sub-criterion divided by the total response of all the sub-criteria in one criterion. On the other hand, percent of cases is response of sub-criteria divided by total respondents involved in the questionnaire. The total percent of cases will over than 100%, since one respondent might have selected more than one sub-criterion.

#### 4.4.1 Appropriate site development

The environment has a maximum threshold in supporting life and human population. Selecting and developing a site that considers the principles of ecology and follows science and land use buildings can potentially reduce its negative impact on the environment, enhance human comfort and provide ease of access to daily activities. Design and development of the site from designers should stay at their optimum capacity and avoid any misuse and overload.

The result shows respondents response varied among sub-criterion from sixteen to thirty seven. Based on the percent of cases, 69.81% of respondents select site selection as the most dominant factors followed by stormwater management (52.83%), site landscaping (50.94%), microclimate (50.94%), public transport (43.40%) and bicycle facility (30.19%). The average of the response is 49.69%, therefore only site selection, stormwater management, site landscaping, and microclimate are significant for criterion of appropriate site development. Furthermore, the sequential order of cost efficiency related to appropriate site development is energy, water, operation and renewals, investment, non - fuel operational and salvage value respectively.

#### 4.4.2 Energy efficiency & conservation

This sub-criterion aims to encourage energy source savings by tailoring how they impact on the environment. Designing building envelope with abundant natural lighting will reduce energy output. Artificial lighting shall be designed with precise calculation according to building requirement. On the other hand, natural cooling needs to be installed with an optimum ventilation system to reduce energy consumption and avoid heat transfer to the atmosphere.

The result shows respondents response varied among sub-criterion from fifteen to thirty four. Based on the percent of cases, 69.15% of respondents select building envelope and ventilation as the two most dominant factors followed by natural lighting (64.26%), low energy elements (54.72%), environmental friendly refrigerant (35.85%), renewable energy (35.85%) and vertical transportation (28.30%). The average of the response is 49.33%, therefore only building envelope, ventilation, natural lighting and low energy elements are significant for criterion of energy efficiency and conservation. The cost efficiency related to energy efficiency and conservation are followed in sequence from energy, operation, and renewals, water, non - fuel operational, investment and salvage value.

#### 4.4.3 Water conservation

Currently, Indonesia requires about 8 million meter cubic of clean water which rises 10 % annually. The urban area experiences water scarcity due to poor gray and black water management. The issue about waste management also results in pollution of water bodies and damage to the environment. Water conservation can minimize water consumption in greater scale to provide a balanced water supply in the future. Several concept designs to reduce the use of water range from rainwater recycling, water fixture selection and optimum landscaping to the use of alternative water source.

The result shows respondents response varied among sub-criterion from eighteen to thirty eight. Based on the percent of cases, 71.70% of respondents select water recycling as the most dominant factor followed by water efficiency landscaping (54.72%), rainwater harvesting (50.94%), water use reduction (41.51%), water fixtures (39.62%) and alternative water resources (33.96%). The average of the response is 48.74%, therefore only water recycling, water efficiency landscaping, and rainwater harvesting are significant for criterion of water conservation. The sequence of cost efficiency related to water conservation is water, energy, non – fuel operational, maintenance and renewals, investment and salvage value.

#### 4.4.4 Material resources & cycle

It aims to optimize material usage to prolong its life cycle through conservation and efficiency. The process started from product development phase, processing, and production, design, and application to the building. The local material shall be used to diminish carbon footprint and ecological imprint which was potentially produced during the manufacturing process.

The result shows respondents response varied among sub-criterion from sixteen to thirty nine. Based on the percent of cases, 73.58% of respondents select environmental-friendly material as the most dominant factor followed by building and material reuse (49.06%), regional material (43.40%), and certified wood (30.19%). The average of the response is 49.06%, therefore only environmental-friendly material, and building and material reuse are significant for criterion of material resources and cycle. Accordingly, cost efficiency related to material resources & cycle follows in sequence from investment, energy, operation and renewals, salvage value, non - fuel operational and water.

#### 4.4.5 Indoor air health & comfort

Indoor air quality affects human health since 90% people spend most of their time indoors. Poor air quality either from the inside (e.g., functional failure of the cooling system) or outside of the building may harm the environment and human health. Passive design implementation and proper maintenance should be managed to mitigate comfort issues and in the long term to increase people productivity. The building also requires maintaining its thermal comfort at 25 °C and 60% of humidity, noise cancellation as well as less chemical evaporation for indoors to protect human health. Lastly, designing a building that has outdoor view may reduce eye fatigue and provides a visual experience to the users.

The result shows respondents response varied among sub-criterion from fourteen to thirty four. Based on the percent of cases, 64.15% of respondents select CO2 monitoring as the most dominant factor followed by thermal comfort (62.26%), tobacco smoke control (52.83%), chemical pollutant reduction (33.96%), acoustic level management (28.03%) and outdoor view (26.42%). The average of the response is 44.65%, therefore only CO2 monitoring, thermal comfort and tobacco smoke control are significant for criterion of indoor air health and comfort. Furthemore, the order of cost efficiency related to indoor air health & comfort is energy, operation, and renewals, investment, non - fuel operational, water and salvage value.

#### 4.4.6 Building environment management

Principally, management relates to planning, organizing, actuating and controlling (POAC). It focuses on human resources as the central part of green building continuity to support the achievement of objectives in other categories. GBCI categorized building environment management into five sub-criteria comprising waste management, proper commissioning, accredited expert monitoring, construction activities management, and occupant survey.

The result shows respondents response varied among sub-criterion from twenty two to thirty. Based on the percent of cases, 56.60% of respondents select waste management as the most dominant factor followed by proper commissioning (54.72%), accredited expert monitoring (54.72%), construction activities management (47.17%), and occupant survey (41.51%). The average of the response is 50.94%, therefore only waste management, proper commissioning and accredited expert monitoring are significant for criterion of building environment management. Cost efficiency related to building environment management follows a sequential order from operation and renewal, energy, non - fuel operational, investment, salvage value, and water. The perception of respondents of the green building rating in Indonesia is summarized in Table 9.

**Table 9:** Perception of respondents of the green building rating in Indonesia.

Criterion	Response		Percent of Cases
	N	Percentage	
Appropriate Site Development			
Site selection	37	23.42%	69.81%
Storm water management	28	17.72%	52.83%
Site landscaping	27	17.09%	50.94%
Microclimate	27	17.09%	50.94%
Public Transport	23	14.56%	43.40%
Bicycle facility	16	10.13%	30.19%
Energy Efficiency & Conservation			
Building envelope	34	18.58%	64.15%
Ventilation	34	18.58%	64.15%
Natural lighting	33	18.03%	64.26%
Low energy elements	29	15.85%	54.72%
Environmental friendly refrigerant	19	10.38%	35.85%
Renewable energy	19	10.38%	35.85%
Vertical transportation	15	8.20%	28.30%
Water Conservation			
Water recycling	38	24.52%	71.70%
Water efficiency landscaping	29	18.71%	54.72%
Rainwater harvesting	27	17.42%	50.94%
Water use reduction	22	14.19%	41.51%
Water fixtures	21	13.55%	39.62%
Alternative water resources	18	11.61%	33.96%
Material Resources & Cycle			
Environmental friendly material	39	37.50%	73.58%
Building and material reuse	26	25.00%	49.06%
Regional material	23	22.12%	43.40%
Certified wood	16	15.38%	30.19%
Indoor Air Health & Comfort			
CO2 monitoring	34	23.94%	64.15%
Thermal comfort	33	23.24%	62.26%
Tobacco smoke control	28	19.72%	52.83%
Chemical pollutant reduction	18	12.68%	33.96%
Acoustic level management	15	10.56%	28.30%
Outdoor view	14	9.86%	26.42%
Building Environment Management			
Waste management	30	21.13%	56.60%
Proper commissioning	29	20.42%	54.72%
Accredited expert monitoring	29	20.42%	54.72%
Construction activities management	25	17.61%	47.17%
Occupant survey	22	15.49%	41.51%

### 4.5 Discussions

Based on the questionnaire result, criteria from green building rating has a varied response from the stakeholders. Respondent needs to acknowledge all sub-criteria if they understand green building rating. Each sub-criteria support the criteria to obtain the available maximum points and bonus points in energy efficiency and conservation criteria. It will determine the building rating (silver, gold, platinum).

Overall, there are sub-criteria that below twenty response except occupant survey criterion in building environment management. Firstly, bicycle facility has the lowest response with 16 response in appropriate site development criteria. The low response may correlate the use of the bicycle for work. In Jakarta, people are used to travel mostly by private vehicles such as motorbike and cars. While others prefer public transportation such as bus or train. Inadequate landscape and inappropriate spatial planning of the city becomes obstacle the use of bicycle to work. From the observation, limited buildings in the capital city have provided proper bicycle parking. When the space available, no bicycle units are using the facility. Consequently, bicycle facility arguably not a mandatory consideration in developing new buildings. The bicycle should be seen as a substitution of non-renewable vehicles. It does not only serve as part of trend and lifestyle but also used in daily commuting as our effort in making the earth green.

Infrastructure is critical to increasing bike users exponentially. Several countries in Europe such as The Netherlands, Denmark, and Germany have previously set up a congenial, friendly infrastructure: bicycle-only parking, public transport integration, comprehensive traffic education and training for cyclists. Providing bicycle facility from building owners may not increase the use of bike users when the supporting infrastructure is not available. Various stakeholders from a government institution, city planners, building owners, community, public transportation experts and many others have to be involved in creating the grand design of sustainability in the capital city. Thus, minimizing the greenhouse gas emission can be achieved.

On the other hand, vertical transportation has the lowest response (15 response) in energy efficiency and conservation criteria. Respondent tends to exclude this sub-component due to common logic that high-rise buildings have to involve lifts for transporting people and goods to designated area. For operation, it may require higher electricity and energy consumption compared to low-rise buildings that use stairs and compact vertical transportation. However, vertical transportation located within the building core is crucial for structural and architectural considerations. Many innovations have taken place in the elevator industry, and the use of technology and advanced artificial intelligent contribute to reduce total energy consumption, operation, and maintenance cost, vibration level and many others (Wong and Li, 2008).

Moreover, certified wood is the least option selected (16 response) by respondent in term of material resources and cycle criteria. It is unknown whether the respondent has limited knowledge of certified wood or simply because it depends on building owner. There are numerous forest certification programs in the world covering a different type of forests with different expiration periods. FAO (n.d) argued that Forest Stewardship Council (FSC) and Programme for the Endorsement of Forest Certification (PEFC) as the two largest international forest certifications. FSC certified 198,996,506 Ha of forest, with 33,646 chain of custody (CoC) certificates (FSC.org). On the other hand, PEFC covers 304 million Ha of forests with more than 19,800 of CoC (PEFC.org).

In Indonesia, wood certification is mandatory and verified by the Ministry of Forestry under the Timber Legality Assurance (SVLK) system. It is a tracking system whose development was made by involving multi-stakeholders to assure the legality of sources from which timber being traded in Indonesia originates (Ministry of Forestry, 2018). SVLK attempt to convince national and international consumers about the timber legality from Indonesia. Ministry of Forestry also aims the system to improve forest governance in Indonesia and to enhance the competitiveness of Indonesia's timber products, to deter illegal logging and illegal timber trading and to elevate community's welfare. Indonesia is the first country in the world to certify timber (Antara, 2013), while other countries have so far handed their timber product certification programs to non-governmental organizations. However, in contrast to a certificate of sustainable forest management, the SVLK certificate merely states that the product has legally valid origins.

Lastly, the outdoor view is the lowest percentage in indoor air health and comfort criteria. The reasons of limited interest in this sub criteria may depend on individual perception. When individual workers are spending time indoors during working hours, connecting outdoors is occasionally neglected due to paperwork focus or limited time for relaxing. Building occupants mostly not have the ability to determine their seating arrangement near the windows or close to the core of buildings. Thus, most of them compensate the lack of outdoor visual by placing natural environment images, plantation, or decorations associated with nature theme and landscape (Berto, 2005; Bringslimark et al., 2011). However, building designers have the competency to create a footprint that incorporates adequate outdoor view for building occupants. A comprehensive study during the initial phase of building development is then required by understanding the physiological of occupant needs. Its attempts to utilize worker performance and on par reduce users stress, multiple health complaints, and sick leave from the minimum access to outdoor view.

#### 4.5.1 Cost efficiency

**Fig. 3:**
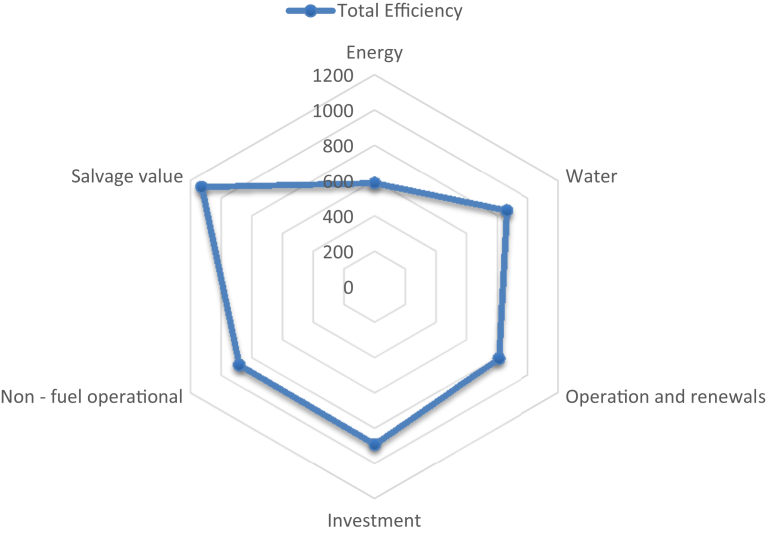
Potential cost efficiency related to six criteria in green building.

All data from respective parts were added up to obtain total cost efficiency that may be produced from the criteria from green building. The lowest value is the most potential cost reduction. Salvage value scores about 1,128 points followed by investment (890 points), non-fuel operational (883 points), water (862 points), operation and renewals (810 points) and energy (586 points). The result shows that energy cost leaving behind other efficiency components. Thus the implementation of green building rating system may reduce energy cost significantly. Another efficiency also from operation and renewals as well as water consumption. The result can be seen in Fig. 3.

## 5 Conclusions

This study investigates stakeholder's perspective to sustainability and green building rating components. The result shows that most of the professionals in this sector prefer environmental issues as the primary concern in developing new building over social and economic aspects of sustainability. Energy efficiency and water conservation are two dominant factors for building owner to implement the green building concept. Moreover, stakeholders consider energy consumption and environmental protection as the primary concern when project life cycle is taking into account to achieve sustainability design. Energy cost argued can be reduced significantly by adopting this type of concept.

Based on the result analysis, respondent experience in sustainable design is about 43.80%, and only 17.10% of them have green building certificate. Despite their awareness of this concept, limited experience from building owner and rewards are the two main barriers to the steady progress of professionals taking the green building certification in Indonesia and the adoption of green building. The respondents argued that government should have more role to accelerate the green building implementation to the country. This findings also supported by Chan et al. (2018), that government-related barriers exists in developing countries to the adoption of green buildings. Policy and regulation of green building should be strengthen by the government by taking into account related stakeholders input such as academics, associations, building owner, contractors to practitioners.

Furthermore, criteria from green building rating have a varied response from the stakeholders. Bicycle facility, vertical transportation, alternative water resources, certified wood and outdoor view, are sub-criteria that has a low response from stakeholders. Although the choices may differ among stakeholders, the current status of green building in the country and building owner authority for decision making are the two reasons not all green building rating criteria selected. Therefore, promoting the concept of green building and its components through socialization, workshop, and knowledge sharing should becomes the priority in the near future.

This research has several limitations. Firstly, the respondent is only considered from Indonesian nationality to generate uniformity of knowledge in green building in the country. The result may be different when the questionnaire survey is taking into account foreign employee or practitioners that work in Indonesia. The study also can be replicated in other countries to compare the level of knowledge, stakeholder interest in the green building project and practitioners taking green building certification. Secondly, the study has a relatively small size of the sample due to limited local experts in green building with extensive experience related to sustainability design. Future research suggested involving not only about building to environment, but about also sustainable buildings to socio-economic development and government policies and regulations.

## Declarations

### Author contribution statement

Mohammed Ali Berawi: Conceived and designed the experiments; Contributed reagents, materials, analysis tools or data; Wrote the paper.

Perdana Miraj, Abdur Rohim Boy Berawi: Analyzed and interpreted the data; Wrote the paper.

Retno Windrayani: Conceived and designed the experiments; Performed the experiments; Analyzed and interpreted the data; Contributed reagents, materials, analysis tools or data.

### Funding statement

This work was supported the United States Agency for International Development (USAID) through the Sustainable Higher Education Research Alliance (SHERA) Program for Universitas Indonesia's Scientific Modeling, Application, Research and Training for City-centered Innovation and Technology (SMART CITY) Project, Grant #AID-497-A-1600004, Sub Grant #IIE-00000078-UI-1.

### Competing interest statement

The authors declare no conflict of interest.

### Additional information

No additional information is available for this paper.
